# Associations between empathy and big five personality traits among Chinese undergraduate medical students

**DOI:** 10.1371/journal.pone.0171665

**Published:** 2017-02-10

**Authors:** Yang Song, Meng Shi

**Affiliations:** English Department, School of Fundamental Sciences, China Medical University, Shenyang North New Area, Shenyang, Liaoning Province, P.R.China; Universidade de Mogi das Cruzes, BRAZIL

## Abstract

**Background:**

Empathy promotes positive physician-patient communication and is associated with improved patient satisfaction, treatment adherence and clinical outcomes. It has been suggested that personality traits should be taken into consideration in programs designed to enhance empathy in medical education due to the association found between personality and empathy among medical students. However, the associations between empathy and big five personality traits in medical education are still underrepresented in the existing literature and relevant studies have not been conducted among medical students in China, where tensions in the physician-patient relationship have been reported as outstanding problems in the context of China’s current medical reform. Thus, the main objective of this study was to examine the associations between empathy and big five personality traits among Chinese medical students.

**Methods:**

A cross-sectional study was conducted in a medical university in Northeast China in June 2016. Self-reported questionnaires including the Interpersonal Reactivity Index (IRI) and Big Five Inventory (BFI) and demographic characteristics were distributed. A total of 530 clinical medical students became our final subjects. Hierarchical regression analysis was performed to explore the effects of big five personality traits on empathy.

**Results:**

Results of this study showed that big five personality traits accounted for 19.4%, 18.1%, 30.2% of the variance in three dimensions of empathy, namely, perspective taking, empathic concern and personal distress, respectively. Specifically, agreeableness had a strong positive association with empathic concern (β = 0.477, P<0.01), and a moderate association with perspective taking (β = 0.349, P<0.01). Neuroticism was strongly associated with personal distress (β = 0.526, P<0.01) and modestly associated with perspective taking (β = 0.149, P<0.01). Openness to experience had modest associations with perspective taking (β = 0.150, P<0.01) and personal distress (β = -0.160, P<0.01). Conscientiousness had a modest association with perspective taking (β = 0.173, P<0.01).

**Conclusion:**

This study revealed that big five personality traits were important predictors of self-reported measures of both cognitive and affective empathy among Chinese medical students. Therefore, individualized intervention strategies based on personality traits could be integrated into programs to enhance empathy in medical education.

## Introduction

Empathy is an essential element in medical practice, which promotes positive physician-patient communication and is associated with improved patient satisfaction, treatment adherence and clinical outcomes [1–3]. Empathy in physicians facilitates more accurate diagnosis and shared decision making [4,5] and patients report higher levels of satisfaction, self-efficacy and reduced emotional distress when they perceive a physician as more empathetic [6]. While the construct of empathy has been construed as an anchoring and adjustment process [7], temporary state [8], or a stable personality trait [9,10], authors have recently conceptualized empathy as a complex multidimensional socio-emotional competency, which consists of both cognitive and affective components [11,12]. In the clinical context, the cognitive component of empathy includes the physician’s ability to understand the patient’s perspective and select counseling and treatment accordingly, whereas the affective component is widely referred to as empathetic concern or sympathy, which includes the ability to recognize the patient’s affect and respond with an appropriate emotion [13,14].

The importance of both the cognitive and affective components of empathy in medical practice has highlighted the necessity of medical schools to create a climate conducive to fostering empathy in medical students [15]. Therefore, recent studies examining empathy in medical students have called greater attention to the potential importance and need for educators to implement curriculum changes designed to promote or slow the decline of empathy among medical students. The majority of previous studies on empathy in medical students have examined their levels of empathy throughout medical school using cross-sectional or longitudinal analyses [15]. Whereas there was a long-standing belief among psychologists that gender was a significant predictor of empathy, with female medical students having higher self-reported empathy scores than their male counterparts [16–20], some studies have reported no significant gender differences in empathy [21,22]. Also, controversies have arisen over the change of students’ empathy level throughout medical education. Some studies have demonstrated that empathy scores increased as students progressed through their medical school studies [16,18], whereas others have found a significant decline in students’ empathy during medical education [17,23,24] and several studies have revealed the stability of medical students’ empathy over time [25,26]. However, there is a scarcity of studies exploring the correlation between empathy and medical students’ psychological variables, in particular their personality traits.

Based on the Five-Factor Model (FFM or the Big Five), personality traits are hierarchically organized into five broad domains, consisting of extraversion, agreeableness, conscientiousness, neuroticism and openness to experience [27]. The FFM has accumulated a compelling body of empirical support and has been validated in many cultures and universally accepted as a basis for comparing, contrasting and integrating diverse sets of personality traits [28,29]. Big five traits have also been linked with behaviors, affective experiences and cognitive processes across the lifespan and cultures [30]. Previous studies have suggested personality traits should be taken into consideration in programs designed to enhance empathy in medical education due to the association found between personality and empathy among medical students. It has been shown that agreeableness and openness to experiences were positively associated with empathy among Portuguese medical students [31,32]. Higher levels of empathy were also found to be correlated with higher sociability and lower aggressive-hostility among first-year medical students [33]. Agreeableness was also shown to be the most important predictor of empathy across cultures among both college-age and adult samples since it is primarily a dimension of interpersonal behavior that represents the quality of social interaction [34,35]. There is documented evidence that agreeableness, which represents the tendency of being altruistic, tender-minded, cooperative, helpful and sympathetic, is responsible for communal and pro-social behavior, or behavior beneficial for others [36,37]. Thus, it seems likely that agreeableness is substantially correlated with empathy, in particular, empathic concern, which reflects other-oriented emotion of helping others in need or feeling responsibility and concerns for the well-being of other people [12]. Also, neuroticism, characterized by an inappropriate level of emotional arousal [38] and negative emotions such as anxiety, depression and self-consciousness, shares many core features with personal distress which reflects the self-centered feelings of discomfort and anxiety when others are distressed [39,40]. Furthermore, openness, especially its facet of attentiveness to inner feelings, may be expected to have positive associations with cognitive empathy, which implies the ability to understand the internal states including the thoughts, feelings, or intentions of other people [9,31]. In addition, conscientiousness was also revealed to have predictive value for empathy [35]. However, as of yet, the associations between empathy and big five personality traits in medical education are still underrepresented in the existing literature and relevant studies have not been conducted among medical students in China, where tensions in the physician-patient relationship have been reported as outstanding problems in the context of China’s current medical reform. Thus, the present study was carried out to examine the associations between empathy and big five personality traits among Chinese medical students.

## Methods

### Study design and sample

This cross-sectional study was conducted in a medical university in Northeast China in June 2016. The sample consists of first-year and second-year undergraduates majoring in clinical medicine from 20 randomly-selected classes. A total number of 575 self-reported questionnaires were distributed in class. 45 invalid questionnaires were excluded, thus a pool of 530 students comprised our final sample with the valid response rate being 92.17%.

Written informed consent was signed by all participants involved in this study according to the Declaration of Helsinki (59th WMA General Assembly, 2008). The study protocol and procedures were conforming to ethical standards and approved by the Ethics Committee of China Medical University.

### Measures

#### Demographic characteristics

Demographic information including age, gender, and academic year was obtained in this study.

#### Measurement of empathy

Empathy was measured with the Interpersonal Reactivity Index (IRI), which is widely used as a measure of empathy disposition in studies among medical students [41]. It consists of three seven-item subscales: (1) perspective taking, which measures spontaneous attempts to adopt others’ perspectives and view things from their point of view; (2) empathic concern, which explores respondents’ feelings of warmth, compassion and concern for others; (3) personal distress, which assesses the personal feelings of anxiety and discomfort evoked by observing others’ negative experience [39]. Each item is scored on a 5-point Likert scale ranging from “does not describe me well”(0) to “describes me very well”(4). Higher scores indicate higher levels of empathy. The Cronbach’s alpha coefficients of perspective taking, empathic concern and personal distress in the present study were 0.784, 0.599 and 0.787, respectively.

#### Measurement of big five personality traits

Big five personality traits were measured using Big Five Inventory (BFI) [37], which consists of 44 items that measure five personality traits, namely, extraversion, agreeableness, conscientiousness, neuroticism and openness. Each item is scored on a 5-point Likert scale ranging from “strongly disagree”(1) to “strongly agree”(5). The Chinese version of the BFI has been widely used and demonstrated adequate reliability and validity [42]. The Cronbach’s alpha coefficients of extraversion, agreeableness, conscientiousness, neuroticism and openness in the present study were 0.705, 0.703, 0.748, 0.721 and 0.774 respectively, which indicate good reliability.

### Statistical analysis

Statistical analyses were performed using SPSS version 17.0 and a two-tailed *P* value of less than 0.05 was considered statistically significant. Descriptive statistics of variables of demographic characteristics, personality and empathy were indicated with mean, standard deviation (SD), number (N) and percentage (%) as appropriate. T-tests were used to compare empathy differences in categorical variables. Pearson’s correlation was conducted to assess correlations between big five personality traits and empathy. Hierarchical regression analysis (HMR) was performed to explore the effects of independent variables on empathy. With scores of empathy being used as dependent variables, two blocks of independent variables were successively entered in the regression model in the following steps: Step 1: demographic characteristics; Step 2: five personality traits. The variance of empathy scores was explained by the relative importance of variables which were retained in the final model as the standardized β. Standardized parameter estimates (the standardized β) were used to compare the magnitudes of the correlations across independent variables. The fit of the model was assessed with the R^2^ value.

## Results

### Description of the participants

Demographic characteristics of the participants and the distribution of three dimensions of the empathy construct are displayed in Table 1. Among the 530 medical undergraduates, 192 (36.2%) were males, while 338 (63.8%) were females, which reflects the reality that females significantly outnumber males in the vast majority of medical universities in China. Their age ranged from 18 to 23. Among them, 303(57.2%) were first-year and 227 (42.8%) were second-year medical students. No significant differences in gender or academic year were observed with regard to three different dimensions of empathy construct (*P*>0.05).

**Table 1:** Demographic characteristics of participants.

Variables	Number	Percent	Perspective taking	Empathic concern	Personal distress
			(Mean±SD)	(Mean±SD)	(Mean±SD)
Gender					
Male	192	36.2%	17.22±3.96	22.23±3.83	12.51±4.18
Female	338	63.8%	17.16±4.09	22.91±3.91	12.72±4.34
t			0.170	-1.929	-0.560
*P*-value			0.865	0.054	0.576
Academic year					
1^st^ year	303	57.2%	17.17±3.98	22.41±3.86	12.33±4.06
2^nd^ year	227	42.8%	17.19±4.12	23.00±3.92	13.06±4.53
t			-0.028	-1.735	-1.933
*P*-value			0.977	0.083	0.054

### Correlation between empathy and big five personality traits

The correlations between big five personality traits and empathy are presented in Table 2. Overall, big five personality traits were strongly correlated with empathy. As is shown in this table, extraversion was positively correlated with perspective taking (*P*<0.01) but negatively correlated with personal distress (*P*<0.01). The traits of agreeableness, conscientiousness and openness were positively correlated with perspective taking (*P*<0.01) and empathic concern (*P*<0.01 for agreeableness and conscientiousness and *P*<0.05 for openness) but negatively correlated with personal distress (*P*<0.01). However, neuroticism was found to show a significantly negative correlation with perspective taking (P<0.01) but a positive correlation with personal distress (*P*<0.01).

**Table 2:** Means, standard deviation (SD) and correlations of continuous variables.

Variables	Mean	SD	1	2	3	4	5	6	7	8
1.Extraversion	24.73	5.00	1							
2.Agreeableness	33.43	5.04	.222**	1						
3.Conscientiousness	29.18	5.31	.216**	.352**	1					
4.Neuroticism	23.24	5.40	-.389**	-.523**	-.497**	1				
5.Openness	34.29	6.08	.397**	.215**	.297**	-.261**	1			
6.Perspective taking	17.18	4.04	.148**	.369**	.275**	-.173**	.253**	1		
7.Empathic concern	22.67	3.89	.051	.381**	.125**	-.037	.088*	.246**	1	
8.Personal distress	12.64	4.28	-.200**	-.248**	-.304**	.531**	-.273**	.003	.019	1

*Significant at the 0.05 level (two-tailed)

** Significant at the 0.01 level (two-tailed).

### Predictors of empathy

Results of the hierarchical multiple regression analysis for empathy were presented in Table 3. The demographic factors of age and gender explained only 0.1%, 1.1% and 1.1% of the variance in the three domains of empathy, namely, perspective taking, empathic concern and personal distress, respectively. However, big five personality traits accounted for 19.4%, 18.1%, 30.2% of the variance in perspective taking, empathic concern and personal distress, respectively. After controlling for age and gender, four personality traits were significantly correlated with empathy. Specifically, agreeableness had a strong positive association with empathic concern (β = 0.477, P<0.01) and a moderate association with perspective taking (β = 0.349, P<0.01). Neuroticism was strongly associated with personal distress (β = 0.526, P<0.01), and modestly associated with perspective taking (β = 0.149, P<0.01). It is worth noting that despite the non-significant correlation between neuroticism and empathic concern, the multiple regression results revealed significant relationship, which may be due to suppression effects. Also, openness to experience had a modest positive association with perspective taking (β = 0.150, P<0.01) and a modest negative association with personal distress (β = -0.160, P<0.01). Conscientiousness had a modest association with perspective taking (β = 0.173, P<0.01).

**Table 3:** The results of hierarchical linear regression analyses.

Variables	Perspective taking (β) (β)		Empathic concern (β)		Personal distress (β)	
	Step1	Step2	Step1	Step2	Step1	Step2
**Step 1**						
Age	-0.031	-0.031	0.066	0.055	0.102*	0.070
Gender	-0.012	-0.034	0.094*	0.059	0.041	0.028
**Step 2**						
Extraversion		0.033		0.016		0.061
Agreeableness		0.349**		0.477**		0.056
Conscientiousness		0.173**		0.076		-0.027
Neuroticism		0.149**		0.261**		0. 526**
Openness		0.150**		0.029		-0.160**
F	0.264	18.044**	2.991	17.745**	2.846	33.890**
R^2^	0.001	0.195	0.011	0.192	0.011	0.312
△R^2^	0.001	0.194	0.011	0.181	0.011	0.302

** *P***<**0.01 (two-tailed)

* *P***<**0.05 (two-tailed).

## Discussion

This study examined the associations between empathy and big five personality traits among Chinese medical students, which has not yet been explored in previous studies. Overall, the results of this study revealed that big five personality traits were important predictors of self-reported measures of empathy among the medical students, which explained up to 19.4%, 18.1% and 30.2% of the variance of the three dimensions of empathy, namely, perspective taking, empathic concern and personal distress, respectively. Specifically, agreeableness was found to have relatively strong associations with empathic concern and perspective taking. This finding is consistent with the results of a recent study which revealed that agreeableness was highly correlated with empathic concern and moderately correlated with perspective-taking [12]. It also supported the findings of previous cross-sectional studies conducted among American [15] and Portuguese medical students [31]. Meanwhile, a cross-cultural study conducted among undergraduates from China, Germany, Spain, and the United States of America has also indicated that agreeableness was the most important personality factor to explain perspective taking and empathic concern [35], though students in China and other East Asian cultures were shown to score lower in agreeableness compared with other samples [35,43]. The strong correlations between agreeableness and perspective taking and empathic concern may be due to the fact that these two subscales of empathy share the other-oriented attributes reflected within the domain of agreeableness. That is, the tendencies toward altruism, tender-mindedness, forgiveness, and helpfulness subsumed within agreeableness [36,37] seem consistent with efforts to reduce interpersonal conflict [44] and maintain intragroup cooperation [45], and contrast a prosocial and communal orientation toward others with antagonism [12,31]. Moreover, agreeableness and empathy have been linked to neurophysiological mechanisms including mirror neurons that enable the interpretation of others’ actions and allow the understanding of others’ emotions, intentions and mental states [46–48]. Behavioral and neuroimaging research has revealed that agreeableness is correlated with empathic accuracy and increased brain activity in two brain regions, namely, medial prefrontal cortex and temporoparietal junction, which are important for empathic processing during emotional perspective taking and recognizing the emotional feeling states of other people [49]. Thus, clinical undergraduates who are more agreeable may be expected to be more empathic, in particular, they would be more likely to understand patients’ perspective or mental state and have more sympathy and compassion for patients’ suffering in their future medical practice.

Neuroticism was found to have a strong positive association with personal distress and a modest positive association with perspective taking. Previous studies on the association between neuroticism and empathy have yielded mixed results, with some showing the absence of an association between these two constructs [31,32,50]. However, this finding replicated results of prior research which have found neuroticism to an important predictor of personal distress [12,35,51]. Previous literature has also documented that the neuroticism factor was positively associated with emotional arousability, which underpins the emotional empathic response [52]. According to FFM, neuroticism is concerned with an individual’s propensity to experience negative emotions such as anxiety, fearfulness and insecurity in relationships [53]. Two of the underlying themes in the neuroticism domain are the experience of negative emotions and the inability to successfully regulate one’s emotional reactions. Meanwhile, personal distress, which reflects the “self-oriented” feelings of personal anxiety, discomfort and unease in tense interpersonal settings [54] and the inability to regulate one’s emotions in an effective way [55], shares those two themes when one is faced with another in distress [12,51]. Thus, the negative emotionality and maladaptive emotion regulation that both neuroticism and personal distress have in essence can explain the strong positive association between these two constructs. With respect to the minimal positive association between neuroticism and perspective taking in the present study, one possible explanation could be that people with higher levels of neuroticism tend to be less emotionally stable and are more likely to experience negative feelings such as anxiety, guilt and depressed mood [56]. However, since individuals higher in neuroticism may be more sensitive to environmental stimulation [57], this trait may facilitate their understanding of others’ emotions or feel more compassion and concern for others’ difficulties.

Openness to experience was found to be positively associated with perspective taking and negatively associated with personal distress, but the correlations were comparatively modest. It has been revealed in prior studies that there were positive associations between openness to experience and empathy among Portuguese medical students [31,32]. Those who have high scores on openness to experience are open to new ways of thinking and changes in their environment, thus having the sensitivity and insightfulness to understand other people and the ability to grasp the emotional and personal conditions of others [58]. This could therefore explain the positive association of openness to experience with perspective taking. Also, the finding of the present study that openness was negatively correlated with personal distress is consistent with that of a recent study conducted in Australia [59]. This might be explained by the fact that people scoring high on the trait of openness tend to be more tolerant of human diversity, more open to various situations, prefer more diverse opinions and take pride in being independent thinkers and seeking out new experiences [59,60]. Thus, they would be less likely to become distressed in response to others’ emotional state or condition. It is also worth noting that conscientiousness was shown to be modestly and positively associated with perspective taking, which is not found in prior research [31,32] except a recent cross-cultural study conducted among undergraduates revealing that conscientiousness was an important personality dimension to explain empathic responding [35]. This may be explained by the fact that individuals with higher levels of conscientiousness tend to manage interpersonal conflicts more effectively and provoke fewer disagreements due to their self-disciplines and responsible behavior [61]. This pattern is in line with the association between dispositional perspective taking and lower levels of hostility and aggression [62] and more constructive relationship behaviors [63].

Additionally, no significant differences in gender or year of medical education were observed in the present study with regard to three different dimensions of the empathy construct. This finding was in line with prior research demonstrating that medical students’ empathy did not change significantly during the first two years of medical education [64], contrary to previous findings that suggested a decline of students’ empathy level in the early years of medical education [65]. This could be explained by the fact that awareness has been raised at all levels in recent years on the importance of empathy as a necessary skill of medical students for providing better patient care and as an integral part of professionalism in their future medical practice. Thus, many medical schools in China have introduced curriculum reforms including more liberal arts courses and earlier exposure to real-life clinical training [18], which may promote students’ empathy in their early years of medical education. However, since the study sample consists of only first-year and second-year medical undergraduates, a sample consisting of other academic years should be enrolled in future studies to confirm the stability of students’ empathy throughout medical education [25,26]. Also, the unexpected absence of significant gender differences in empathy was inconsistent with the common belief that female medical students have higher levels of empathy than their male counterparts [16–20], but in line with several studies conducted among Asian medical students suggesting little or no gender difference in empathy [21,22]. This may be due to the fact that female teachers have been outnumbering male counterparts in the classrooms from kindergarten to higher education, which could make students miss out on sufficient role modeling given by male teachers and reduce the opportunities to address some of the gender gap issues [66]. Since they spend more time with teachers in school than with parents at home when they are awake, students’ psychological characteristics and behaviors can be greatly influenced by their teachers [67]. Nevertheless, findings of the present study also lead us to conclude that other important variables beyond gender, age and academic year should be considered in future studies to explain the empathy levels of medical students.

Findings of this study have potential implications for the design of training programs and curriculum to enhance students’ empathy in medical education. Since personality-targeted interventions have gained increasing popularity in health promotion research and practice [68], it might be feasible to enhance empathy of medical students by providing training programs and curriculum designed to raise medical students’ awareness of the correlations between personality traits, empathy and outcomes related to patient care and satisfaction, and how their unique constellation of personality traits may enhance or impinge upon their empathy and doctor-patient interaction [69]. Also, it might be a plausible option to assess students’ personality traits to select the most suitable applicants in the admission process to medical schools [31]. Furthermore, early exposure to clinical practice should be taken into consideration by medical school authorities, thus providing medical students more opportunities to empathize with patients’ needs [18], to gain insight into the impacts that different personality traits may deliver on physician-patient interactions in the real-world clinical settings, and to promote positive physician-patient relationship.

One major limitation of the present study was its cross-sectional design so that no causal relationship among study variables could be inferred from the findings. Thus, longitudinal studies are recommended to establish causality and the reciprocal relations that the variables in the present study might have with each other over time. Second, the self-reported measures of both personality and empathy could introduce response bias. Third, the use of Big Five Inventory could only assess the Big Five domains rather than more specific facet traits within each domain. Thus, further studies using the more comprehensive Revised NEO Personality Inventory would contribute to more refined conclusions about the correlations between personality traits and empathy. Fourth, given the study sample, to what extent these findings could be generalized to medical schools in other nations should be confirmed by further studies. However, in spite of the limitations, strengths of the present study included the large sample size and high response rate. More importantly, this research extended the existing literature on empathy by examining its correlations with the big five personality traits, which has not yet been explored in previous studies among clinical medical students in China where tensions in the physician-patient relationship have been reported as a major social problem.

## Conclusion

This study explores the associations of different domains of empathy with the big five personality traits among Chinese medical students. Results of this study revealed that big five personality traits were strongly correlated with self-reported measures of empathy among Chinese medical students, contributing significantly to the variance of both cognitive and affective empathy. Specifically, agreeableness had relatively strong associations with empathic concern and perspective taking. Neuroticism was strongly associated with personal distress, and modestly associated with perspective taking. Openness to experience was modestly associated with perspective taking and personal distress. Conscientiousness also had a modest and positive association with perspective taking. Therefore, individualized intervention strategies based on personality traits could be integrated into programs to enhance empathy in medical education.

## Supporting information

S1 Dataset The S1 Dataset includes the data concerning the study variables of the subjects in the present study. (SAV)
